# Differential Susceptibility and Vulnerability of Brain Cells in C57BL/6 Mouse to Mitochondrial Dysfunction Induced by Short-Term Cuprizone Exposure

**DOI:** 10.3389/fnana.2020.00030

**Published:** 2020-06-09

**Authors:** Mengyi Luo, Maomao Deng, Zijia Yu, Yi Zhang, Shuqin Xu, Shengping Hu, Haiyun Xu

**Affiliations:** ^1^The Mental Health Center, Shantou University Medical College, Shantou, China; ^2^Department of Forensic Medicine, Shantou University Medical College, Shantou, China; ^3^Department of Human Anatomy, Shantou University Medical College, Shantou, China; ^4^School of Psychiatry, Wenzhou Medical University, Wenzhou, China

**Keywords:** cuprizone, ^1^H-MRS, mitochondrial dysfunction, susceptibility, vulnerability, brain cells

## Abstract

Cuprizone (CPZ) is a chemical chelator toxic to mitochondria of cells. While inducing oligodendrocyte (OL) loss and demyelination, CPZ caused no fatal damage to the other brain cells (neurons, astrocytes, and microglia) in previous studies, suggesting differential susceptibility and vulnerability of brain cells to the CPZ intoxication. To demonstrate this interpretation, C57BL/6 mice were fed rodent chow without or with CPZ (0.2%, w/w) for 7 days. One day later, mitochondrial function of brain cells was assessed by proton magnetic resonance spectroscopy (^1^H-MRS) and biochemical analysis. Another batch of mice were processed to localize the CPZ-induced damage to mitochondrial DNA, label brain cells, and identify apoptotic cells. Compared to controls, CPZ-exposed mice showed significantly lower levels of *N*-acetyl-L-aspartate, phosphocreatine, and ATP detected by ^1^H-MRS, indicating mitochondrial dysfunction in brain cells. Susceptibility analysis showed an order of OLs, microglia, and astrocytes from high to low, in terms of the proportion of 8-OHdG labeled cells in each type of these cells in corpus callosum. Vulnerability analysis showed the highest proportion of caspase-3 positive cells in labeled OLs in cerebral cortex and hippocampus, where neurons showed no caspase-3 labeling, but the highest proportion of 8-OHdG labeling, indicating a lowest vulnerability but highest susceptibility to CPZ-induced mitochondrial dysfunction. Immature OLs, microglia, and astrocytes showed adaptive changes in proliferation and activation in response to CPZ-exposure. These data for the first time demonstrated the CPZ-induced mitochondria dysfunction in brain cells of living mouse and specified the differential susceptibility and vulnerability of brain cells to the CPZ intoxication.

## Introduction

Cuprizone (CPZ) is a chemical chelator toxic to mitochondria of cells (Suzuki, 1969; Hoppel and Tandler, 1973). It was shown to reduce activity of monoamine oxidase and copper-dependent enzymes such as cytochrome c oxidase and SOD (Kesterson and Carlton, 1971; Venturini, 1973; Xu et al., 2009; Xuan et al., 2014). CPZ also inhibited functions of complexes I-IV of the respiratory chain in mitochondrion (Pasquini et al., 2007; Millet et al., 2009; Acs et al., 2013). Moreover, CPZ intoxication led to megamitochondria in liver cells and oligodendrocytes (OLs) of mouse (Suzuki, 1969; Acs and Komoly, 2012).

Previous studies have reported that feeding C57BL/6 mice with a 0.2% CPZ-supplemented diet for 4–6 weeks induces acute demyelinating lesions followed by spontaneous remyelination in brains of subjects (Hiremath et al., 1998; Matsushima and Morell, 2001). As such, the CPZ-exposed mouse has been extensively used as an animal model for demyelination and remyelination research (Praet et al., 2014). In addition to demyelination and oligodendrocytes (OLs) loss in the CPZ-exposed mouse, astrocyte proliferation and microglia activation are also prominent in the lesion sites of the mouse brain (Hiremath et al., 1998; Matsushima and Morell, 2001; Stidworthy et al., 2003; Remington et al., 2007; Zhang et al., 2018). An *in vitro* study, however, showed that mature OLs were only ones that were most affected by CPZ, while the other glial cells including microglia, astrocytes, and oligodendrocyte progenitor cells (OPCs), were not or only marginally affected (Bénardais et al., 2013). The above findings suggest different responses of brain cells to CPZ intoxication. That is, the susceptibility and vulnerability of different types of brain cells to CPZ intoxication may be cell-specific. Substantiating this inference was the aim of the present study and is of specific relevance to the pathogenesis of some neuropsychiatric diseases such as multiple sclerosis (MS) and schizophrenia, which involve in a mitochondrial dysfunction mechanism (Mao and Reddy, 2010; Ni and Chung, 2020). As for MS, mitochondrial DNA defects, defective mitochondrial enzyme activities, and deficient mitochondrial DNA repairing activity are important contributors to the development and progression of MS lesions (Mao and Reddy, 2010). Regarding schizophrenia, brain bioenergetic deficits in the production of adenosine triphosphate (ATP) and alterations in mitochondrial size and density were reported in schizophrenia patients (Gonçalves et al., 2015; Sullivan et al., 2018; Ni and Chung, 2020). Also, mitochondrial deficit, altered redox balance and chronic low-grade inflammation were evident in the patients (Rajasekaran et al., 2015).

To provide experimental evidence that different types of brain cells have distinct susceptibility and vulnerability to mitochondrial dysfunction induced by CPZ, a short-term (7-day) CPZ exposure paradigm was applied to C57BL/6 mice in this study. Within this short-term period, CPZ exposure caused no demyelination as reported in previous studies (Hesse et al., 2010; Tezuka et al., 2013), but mitochondrial dysfunction already occurred in brain cells (Xuan et al., 2014). This relatively mild toxic condition enables the measurements on susceptibility and vulnerability of brain cells to mitochondrial dysfunction to be done with fewer confounders in absence of demyelination. The second feature of this study is the application of a non-invasive neuroimaging technique of proton magnetic resonance spectroscopy (^1^H-MRS) to assess mitochondrial functions of brain cells in prefrontal cortex (PFC) and caudate putamen (CPU) of living mice, two brain regions sensitive to CPZ intoxication (Yang et al., 2009). This non-invasive technique has been employed in clinical studies for patients with different types of mitochondrial diseases and revealed the most common metabolic brain abnormalities of decreases in *N*-acetylaspartate (NAA) and phosphocreatine (PCr), as well as accumulation of lactate (Bianchi et al., 2003; Xu et al., 2016). The third advantage is the *in situ* assessment of the CPZ-induced mitochondrial oxidative stress in each type of brain cells using cell-specific antibodies (against NeuN, GST-pi, GFAP, or iba-1) and the antibody against 8-hydroxy-2′-deoxyguanosine (8-OHdG), which is regarded as a biomarker for oxidative stress in cells (Kujoth, 2005; Ma et al., 2011). After double immunofluorescent staining of brain sections with these antibodies, the susceptibilities of various brain cells were compared. For vulnerability comparison, the aforementioned cell-specific antibodies and the antibody against caspase-3, a protein required for the end stage of apoptosis, were used.

## Materials and Methods

### Animals

A total of 28 male C57BL/6 mice in two batches were used in this study. The mice were 6 weeks old when purchased from the Laboratory Animal Center of Southern Medical Laboratory (Guangzhou, China). The mice were housed in groups (4–6 mice/cage) under standard laboratory conditions with a 12-h light/dark cycle, constant room temperature of 23.0 ± 1.0°C, and relative humidity of 50–60%. Cage bedding was changed every other day. All the mice were acclimatized for 7 days under the condition before proceeding to the experiment procedures, which were in accordance with the guidelines set up by the Animal Care and Use Committee of Shantou University Medical College and approved by the committee.

The mice were randomly assigned into either the control group (CNT), in which mice received a standard rodent chow (the main ingredients include: tap water ≤ 10%, crude protein ≥ 18%, crude fat ≥ 4%, crude fiber ≤ 5%, ash ≤ 8%, lysine ≥ 8.2%, methionine ≤ 0.53%, calcium 1.0–1.8%, phosphorous 0.6–1.2%, percentage by weight; Wan Ka Hing Biotechnology Limited, Wuhan, China); or the CPZ group, in which mice consumed the rodent chow containing CPZ at 0.2% (w/w) for 7 days. The first batch (*n* = 8–2/group; the data of two mice in each group were not included for unsuccessful scanning) of mice were used for the assessment of mitochondrial function by means of ^1^H-MRS and biochemical analysis while the second batch (*n* = 6/group) was used for morphological analyses to compare the susceptibility and vulnerability of each type of brain cells to the CPZ-induced mitochondrial damage by employing immunohistochemical and immunofluorescent staining techniques.

### ^1^H-MRS Procedures

As described in previous studies (Xuan et al., 2014; Orije et al., 2015), mice were anesthetized with 5% isoflurane in oxygen delivered via a mask. Body temperatures of mice were recorded and maintained at 36–37°C using a water-heated animal blanket. *In vivo* MR imaging and spectra were acquired using a 7.0 T horizontal DriveDrive 2 MR system (Agilent Technologies, United States) with a 160-mm bore and 400 mT/m actively shielded gradient coil. A dedicated animal brain surface coil (Varian Medical Systems, Inc., Palo Alto, CA, United States) was used as the radio frequency transmitter and the signal receiver. The volumes of interest (VOIs) were positioned in PFC (1.5 × 3.6 × 2.5 mm) and CPU (2.5 × 2.5 × 2.0 mm), but not in CC, which is too small in volume to be a VOI of ^1^H-MRS. Localized shimming was performed automatically for the VOIs to yield a water spectrum width of 15–20 Hz. An ultrashort echo time stimulated echo acquisition pulse sequence was used for the acquisition of proton spectra in the VOIs (TR = 5000 ms, TE = 13.62 ms, dummy scan = 2, real points = 4096, spectral width = 4006 Hz; slice thickness = 10 mm, gap = 0 mm, scan time = 26 min). Water suppression was performed with variable pulse power and optimized relaxation delays.

*In vivo* proton spectra were analyzed using the LCModel as described in previous studies (Provencher, 2001; Orije et al., 2015). A total of 17 metabolites were included and their signal intensities were processed with water scaling for metabolite quantification. In addition to individual metabolite, the sums of some metabolites (NAA + NAAG, Glu + Gln, GPC + PCh, and Cr + PCr) were also estimated.

### Measurement of ATP Levels in Brain Tissue

After ^1^H-MRS, the mice were euthanized with sodium pentobarbital (80 mg/kg, i.p.) and decapitated. The brain was removed from the skull and the brain regions of PFC and CPU were dissected out quickly and frozen in liquid nitrogen until use for ATP measurement which was performed with an ATP Assay Kit (S0026; Beyotime Biotechnology, Shanghai, China) following the operation steps detailed by the manufacturer as follows: (1) sample preparation: the tissue sample was homogenized in the 1 × ATP detection sample buffer provided in the assay kit. After centrifugation (12,000 g, 4°C) for 5 minutes, the supernatant was collected and diluted with the buffer in a pre-chilled polypropylene tube on ice. (2) prepared ATP detection standard solutions by diluting the 5 mM ATP solution in 1 × ATP detection sample buffer in wells A-G. The final ATP concentrations in the wells were in the range of 5 nM –5 mM at the same volume of 200 μL. (3) performing assay: added 100 μL freshly prepared reaction mixture, which was consisted of 1 × ATP detection assay buffer, Dd-Luciferin and Luciferase, to wells on a 96 -wells plate, and kept the plate at room temperature (24°C) for 3 min. Added 20 μL of 1 × ATP detection sample buffer to two blank wells, ATP detection standard to wells A-G (standard wells), and sample solution to designated wells on the plate. Cover the plate with the plate cover and incubated at room temperature for 5–10 min. Removed the plate cover and read the luminescence in a Tecan microplate reader (M20Pro, Tecan Trading AG, Switzerland). Calculated the ATP concentration in each sample after obtaining the average luminescence of blank, standard, and sample wells and subtracting the average luminescence of blank wells from the average luminescence of standard and sample wells. The ATP concentrations of all samples were within the standard curve of the assay.

### Immunofluorescent Staining

Twenty-four hours after the last CPZ-feeding day, the second batch (*n* = 6/group) of mice were euthanized with sodium pentobarbital (80 mg/kg, i.p.) and perfused through the ascending aorta with 0.01M phosphate-buffered saline (PBS; pH7.4), followed by 4% paraformaldehyde in the PBS. The brains were removed and post-fixed overnight in the same fixative, followed by cryoprotection in 30% sucrose for 24 h. Then each brain sample was cut into two hemispheres for immunofluorescent staining as described below.

Each brain hemisphere was sectioned coronally (25 μm) using a cryostat microtome (Leica CM1850). Serial sections were picked up and collected into PBS contained wells of a six-well polyethylene plate in order, starting 2.71 anterior of bregma and finishing 2.12 posterior of bregma by referring to the Mouse Brain (C57BL/6J) in Stereotaxic Coordinates (Franklin and Paxinox, 1997). This range covers the regions of interest (ROI) PFC, CPU, dorsal hippocampus, and CC, among the other brain structures. In this way, each well contained equal numbers (one sixth) of tissue sections from a brain sample (about 33–34 sections) used for immunofluorescent labeling of only one target antigen, i.e., sections in each well of a plate were used for a specific antibody-antigen labeling.

The free-floating sections were washed with PBS for 10 min × 3. Then the sections were incubated with donkey serum (10%; Jackson, West Grove, PA, United States) for 60 min at room temperature. Subsequently, a primary antibody [mouse monoclonal anti-8-OHdG (1:500; Abcam, Cambridge, United Kingdom), rabbit polyclonal anti-NeuN (1:1000; Abcam, Cambridge, United Kingdom), rabbit polyclonal anti-GFAP (1:1000; Abcam, Cambridge, United Kingdom), rabbit polyclonal anti-GST-pi (1:500; Enzo Life Sciences, Germany), or rabbit polyclonal anti-Iba-1 (1:500; Wako Pure Chemical Corporation, Japan)] was added into the blocking solution and incubated overnight at 4°C, followed by incubation at room temperature for 30 min. After rinsed for 10 min × 3 in PBS, sections were incubated with DyLight 488–conjugated donkey anti-mouse antibody (1:200; Jackson, West Grove, PA, United States) or Alexa Fluor 594–conjugated donkey anti-rabbit antibody (1:400; Jackson) in PBST at room temperature for 90 min. After rinsed in PBST, the sections were mounted onto slides and covers-slipped using Fluoro shield Mounting Medium with DAPI (Abcam, Cambridge, United Kingdom). The slides were preserved under 4°C and shielded from light. Immunofluorescence was observed and recorded using a fluorescence microscope (Zeiss InstrumentsInc., Oberkochen, Germany).

The double immunofluorescent staining with antibodies to caspase-3 and APC was done following a similar procedure as described above. The primary antibodies used were rabbit polyclonal anti-caspase-3 (1:100; Abcam, Cambridge, United Kingdom) and mouse monoclonal anti-APC (1:200; Merck Millipore, Darmstadt, Germany). The secondary antibodies were DyLight 488–conjugated donkey anti-mouse antibody and Alexa Fluor 594–conjugated donkey anti-rabbit antibody from the same company (Jackson).

Secondary antibody only controls and isotype IgG antibody controls were performed to ensure that the observed staining was due to the binding of the antibody to the desired antigen and not to some general unspecific binding of the immunoglobulin to the tissue. No positive immunostaining was observed on brain sections in the controls (data not shown).

### Image Analysis

The immunofluorescent stained cells in the ROIs of CC, CA3 area of dorsal hippocampus, and PFC were counted following a stereological principle in order to minimize selection bias. Each group had six mice; one sixth of each mouse brain sections (in one well) were subjected to immunofluorescent labeling for one target antigen. After finishing immunofluorescent staining procedures, brain sections were observed quickly under the fluorescence microscope to find the sections encompassing PFC between bregma 2.71–1.70 and the sections encompassing the dorsal hippocampus and CC between bregma −1.28 – −2.12 (Franklin and Paxinox, 1997). Then the selected sections (6 PFC sections and 5 hippocampus sections per mouse) were scrutinized under the microscope by one of the authors. Only three of these 6 or 5 sections were photographed. We recognize that our imaging and quantification procedures may allow for bias in these measurements (Brown, 2017). Three microphotographs were taken from each selected section at same locations under a same condition by another author. The data (each group had 6 × 3 × 3 microphotographs) were automatically quantified by Image-Pro Plus 6.0 software (Media Cybernetics, Rockville, MD, United States) and analyzed by one the authors who was blind to identity which animal group the sections came from. The cell density was expressed as the number per square millimeter.

### Statistical Analyses

The software Excel 2007 and SPSS 19.0 were used for data analysis. All data are expressed as the mean ± standard deviation (SD). Normality tests were performed for all data using the Shapiro–Wilk test. For the non-parametric data (expressed as%), Wilcoxon-Wilcox multiple comparisons or Mann–Whitney *U* tests were made. For comparison of parametric data from the CNT and CPZ groups, Student *t*-test was done. A level of *p* < 0.05 was considered statistically significant.

## Results

### CPZ-Induced Mitochondrial Dysfunction in Mouse Brain Cells

**FIGURE 1:**
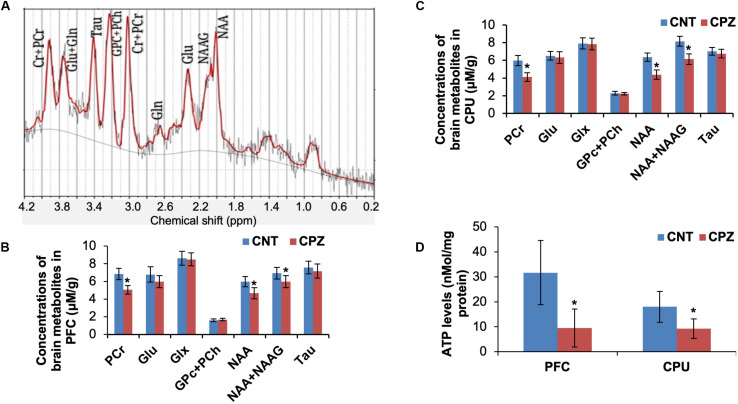
**(A)** A representative proton magnetic resonance spectrum obtained from a VOI (PFC) of a control mouse. **(B)** The bar chart showing the quantitative analysis of brain metabolites in PFC of mice in control and CPZ groups detected by ^1^H-MRS. **(C)** The bar chart showing the quantitative analysis of brain metabolites in CPU of mice in control and CPZ groups detected by ^1^H-MRS. **(D)** The bar chart showing the quantitative analysis of ATP levels in PFC and CPU of mice in control and CPZ groups. Data are expressed as means ± SD (*n* = 6/group). *p < 0.05. The comparisons were made between CNT and CPZ groups by performing *t*-test.

The mitochondrial function of brain cells in living mice was measured by means of ^1^H-MRS. Of the measured brain metabolites (Figure 1A), NAA, total NAA [NAA + *N*-acetylaspartyl-glutamate (NAAG)] and PCr levels were significantly decreased in CPU and PFC of CPZ group mice relative to CNT group (Figures 1B,C) while the others were comparable between the two groups. After the ^1^H-MRS scanning, ATP levels in the two brain regions were analyzed. The results indicate that CPZ group had significantly lower ATP levels in these two brain regions relative to CNT group (Figure 1D).

### CPZ-Induced Oxidative Stress Damage to Glial Cells in Corpus Callosum

**FIGURE 2:**
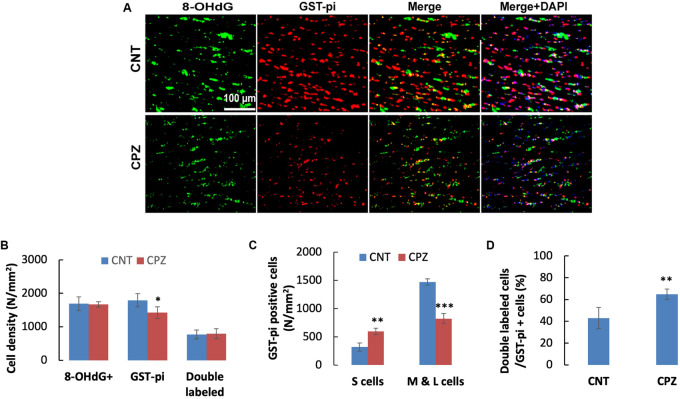
**(A)** Representative photographs showing cells labeled by the antibody to 8-OHdG, antibody to GST-pi, and double labeled cells by the two antibodies. **(B–D)** Bar charts showing the statistical analysis results of the immunofluorescent staining. Data are expressed as means ± SD (*n* = 6/group). *p < 0.05, **p < 0.01, ***p < 0.0001. The comparisons were made between CNT and CPZ groups by performing t-test or chi-square test (percentage of double labeled cells in GST-pi^+^ cells).

Double immunofluorescent staining was performed with the brain sections using antibodies to 8-OHdG and GST-pi. The GST-pi positive cells in CC of mice are shown in Figure 2A. Compared to those in CNT group, the labeled profiles in the CC of CPZ-exposed mice look smaller. Therefore, they were classified into two sub-groups, i.e., small (<100 pixels) and middle-large (101–10,000 pixels) ones, followed by cell counting for each type of them. The statistical analysis on the quantitative data of the labeled profiles revealed that CPZ group showed a lower density of GST-pi positive profiles compared to CNT group, indicating OLs loss occurred in CPZ-treated mice, although the two groups had comparable densities of 8-OHdG positive cells and double labeled cells (Figure 2B). Furthermore, CPZ group showed lower density of middle-large OLs, but a higher density of small ones as compared to CNT group (Figure 2C), indicating that some mature (middle-large size) OLs died while immature (small size) OLs proliferated in response to CPZ intoxication. The percentage of double labeled cells in the GST-pi positive cells in CPZ-exposed mice, however, was significantly higher than that in CNT group (Figure 2D).

**FIGURE 3:**
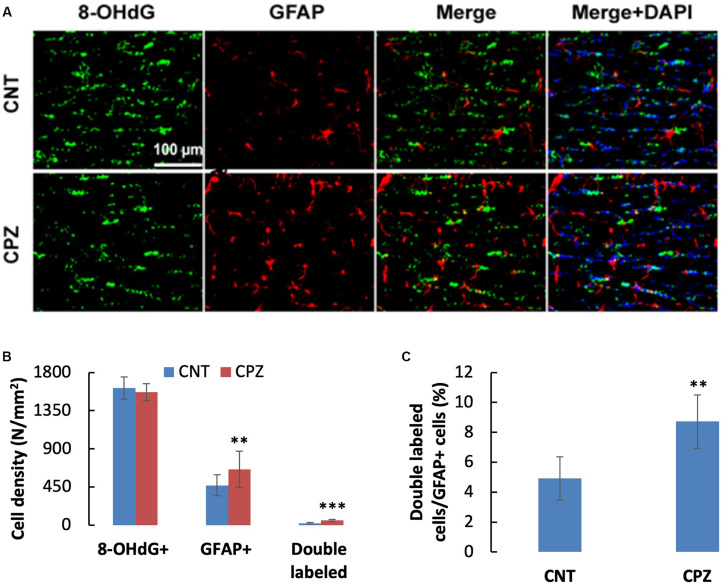
**(A)** Representative photographs showing cells labeled by the antibody to 8-OHdG, antibody to GFAP, and double labeled cells by the two antibodies. **(B,C)** Bar charts showing the statistical analysis results of the immunofluorescent staining. Data are expressed as means ± SD (*n* = 6/group). **p < 0.01, ***p < 0.0001. The comparisons were made between CNT and CPZ groups by performing *t*-test or chi-square test (percentage of double labeled cells in GFAP^+^ cells).

To assess the CPZ-induced oxidative damage to astrocyte mtDNA, double immuno-fluorescent staining was performed with the brain sections of mice using the antibodies to 8-OHdG and GFAP. As shown in Figure 3A, CPZ mice showed many more GFAP positive cells than the mice in CNT group. This observation was confirmed by statistical analysis on the numbers of the labeled cells, which revealed that CPZ exposure significantly increased the density of GFAP positive cells in CC of mice but had no effect on the density of 8-OHdG positive cells (Figure 3B). Also, CPZ-exposure significantly increased the density of double labeled cells in CC of mice (Figure 3B) and elevated the percentage of double labeled cells in the GFAP positive cells (Figure 3C).

**FIGURE 4:**
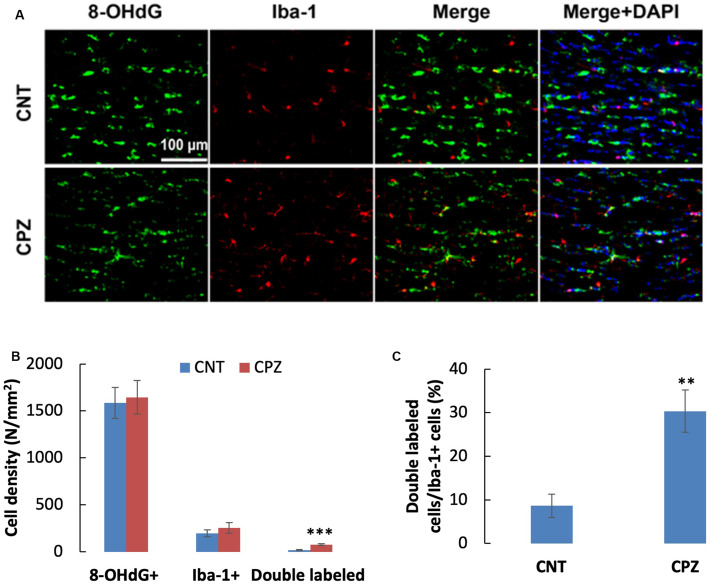
**(A)** Representative photographs cells labeled by the antibody to 8-OHdG, antibody to Iba-1, and double labeled cells by the two antibodies. **(B,C)** Bar charts showing the statistical analysis results of the immunofluorescent staining. Data are expressed as means ± SD (*n* = 6/group). **p < 0.01, ***p < 0.0001. The comparisons were made between CNT and CPZ groups by performing *t*-test or chi-square test (percentage of double labeled cells in iba-1^+^ cells).

To assess the CPZ-induced oxidative damage to microglia mtDNA, double immuno-fluorescent staining was performed with the brain sections of mice using the antibodies to 8-OHdG and iba-1 (a biomarker of mature microglia/macrophages). As shown in Figure 4A, CPZ mice seemed to have many more iba-1 positive cells compared to the mice in CNT group. This observation was in line with the statistical analysis on the numbers of the labeled cells, which revealed that CPZ exposure marginally (*p* = 0.063) increased the density of iba-1 positive cells in CC of mice but had no effect on 8-OHdG positive cells (Figure 4B). Also, CPZ-exposure significantly increased the density of double labeled cells in CC of mice (Figure 4B) and elevated the percentage of double labeled cells in the iba-1 positive cells (Figure 4C).

### CPZ-Induced Oxidative Stress Damage to Neurons in PFC and Hippocampus

**FIGURE 5:**
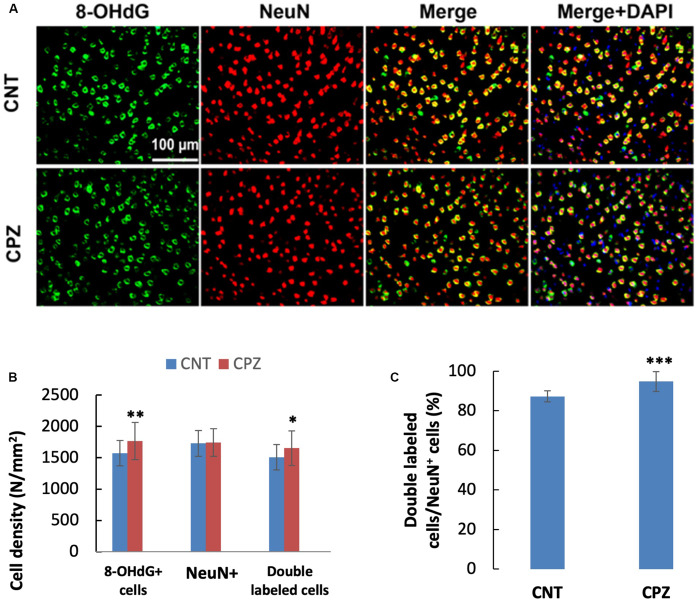
**(A)** Representative photographs showing cells labeled by the antibody to 8-OHdG, antibody to NeuN, and double labeled cells by the two antibodies. **(B,C)** Bar charts showing the statistical analysis results of the immunofluorescent staining. Data are expressed as means ± SD (*n* = 6/group). *p < 0.05, **p < 0.01, ***p < 0.0001. The comparisons were made between CNT and CPZ groups by performing *t*-test or chi-square test (percentage of double labeled cells in NeuNi^+^ cells).

**FIGURE 6:**
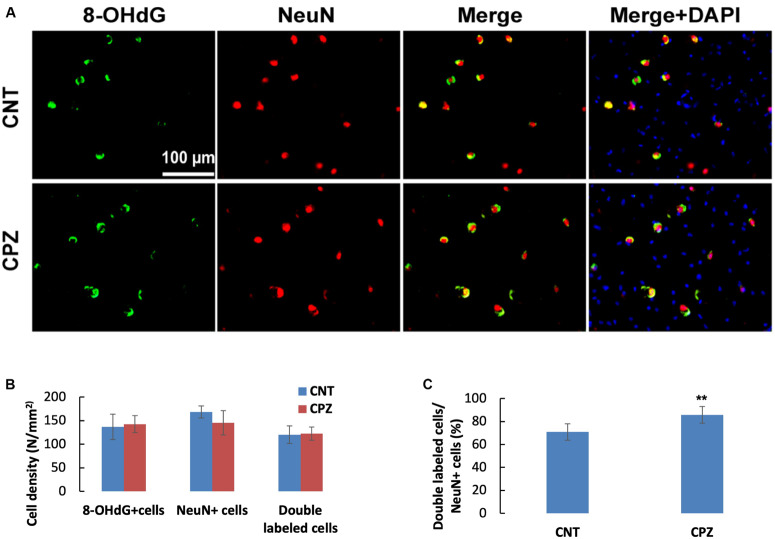
**(A)** Representative photographs showing cells labeled by the antibody to 8-OHdG, antibody to NeuN, and double labeled cells by the two antibodies. **(B,C)** Bar charts showing the statistical analysis results of the immunofluorescent staining. Data are expressed as means ( SD (n ( 6/group). **p < 0.01. The comparisons were made between CNT and CPZ groups by performing *t*-test or chi-square test (percentage of double labeled cells in NeuNi^+^ cells).

To assess the CPZ-induced oxidative damage to neuronal mtDNA, double immunofluorescent staining was performed with the brain sections of mice using the antibodies to 8-OHdG and NeuN (neuronal nuclear antigen). As shown in Figure 5A, the majority of NeuN labeled cells are 8-OHdG positive. The statistical analysis on the numbers of the labeled cells revealed that the CNT and CPZ groups had comparable densities of NeuN positive cells in PFC, but CPZ group presented a significantly higher densities of 8-OHdG positive cells and of double labeled cells (Figure 5B). Also, CPZ-exposure significantly increased the percentage of double labeled cells in the NeuN positive cells (Figure 5C). In CA3 area of the hippocampus, the CNT and CPZ groups were comparable in terms of densities of NeuN positive cells, 8-OHdG positive cells, and of double labeled cells. However, CPZ exposure significantly increased the percentage of double labeled cells in the NeuN positive cells as compared to CNT group (Figure 6).

### Comparison on Susceptibilities of Brain Cells to CPZ-Induced Oxidative Stress

As mentioned above, 8-OHdG is regarded as a biomarker of mtDNA oxidative damage. Therefore, the percentage of 8-OHdG positive cells in each type of brain cell including neuron, astrocyte, OL, and microglia may serve as a reliable parameter for the *in situ* assessment of mitochondrial oxidative stress in the cells. Following this rationale, we compared the percentages of 8-OHdG positive cells in neurons and glial cells in brains of CPZ-exposed mice. As shown in Table 1, 85.74% of neurons in the hippocampus of CPZ-exposed mice were 8-OHdG positive, which is the highest in all cell populations. The percentages of 8-OHdG positive cells in labeled OLs, microglia, and astrocytes were 64.84, 30.36, and 8.73%, respectively, suggesting a high-to-low order of susceptibilities of these glial cell populations to CPZ-induced oxidative stress. Although mice in CNT group also showed various percentages of 8-OHdG positive cells in each type of brain cells, they were significantly lower compared to corresponding counterpart, suggesting that the CPZ-induced mitochondrial dysfunction enhanced the susceptibility of brain cells to oxidative stress resulted from anesthesia (discussion later).

**TABLE 1:** Differential susceptibilities of brain cells to CPZ-induced mitochondrial oxidative stress.

	8-OHdG^+^ in NeuN^+^ cells (%)	8-OHdG^+^ in GST-pi^+^ cells (%)	8-OHdG^+^ in Iba-1^+^ cells (%)	8-OHdG^+^ in GFAP^+^ cells (%)	*P*-value
CNT	70.87	43.00	8.67	4.91	<0.001
CPZ	85.74	64.84	30.86	8.73	<0.001
*P*-value	<0.001	<0.001	<0.001	<0.01	

*P-values resulted from chi-square test. n = 6 per group.*

### CPZ Short-Term Exposure Induces Apoptosis in OLs, but Not in Neurons

**FIGURE 7:**
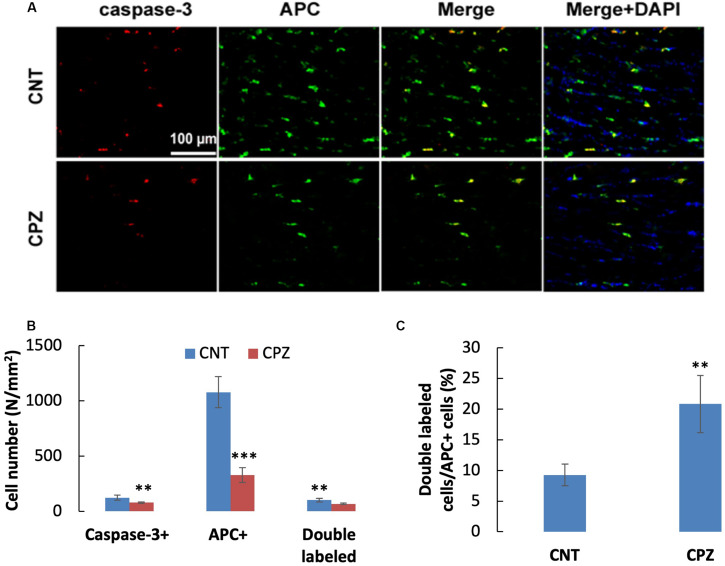
**(A)** Representative photographs showing cells labeled by the antibody to caspase-3, antibody to APC, and double labeled cells by the two antibodies. **(B,C)** Bar charts showing the statistical analysis results of the immunofluorescent staining. Data are expressed as means ( SD (n ( 6/group). **p < 0.01, ***p < 0.0001. The comparisons were made between CNT and CPZ groups by performing *t*-test or chi-square test (percentage of double labeled cells in APC^+^ cells).

A previous study demonstrated that OLs death occurs days after initiation of the CPZ diet and is obvious weeks before demyelination. In the early stage, but not the late stage, dying OLs express activated caspase-3 (Hesse et al., 2010). In the present study, CPZ exposure for 7 days significantly decreased the density of middle-large GST-pi positive cells in CC but did not change the density of NeuN positive cells in PFC and hippocampus of mice though majority of the neurons were 8-OHdG positive. The results suggest that CPZ-induced oxidative stress was fatal for the middle-large OLs, but it was not toxic enough to cause neuronal apoptosis. To confirm this suggestion, we performed single and double immunofluorescent staining using the antibody to caspase-3, and antibodies to caspase-3 and APC (adenomatous polyposis coli protein, a biomarker of mature OLs). As expected, caspase-3 positive cells were found in CC, but not in PFC and hippocampus of mice (data not shown). As shown in the zoomed excerpts, positive caspase-3 staining (red) presented at various sizes smaller than the positive APC profiles (green). A few of them overlapped with the APC profiles, but none of them overlapped with the nuclei (blue) of glia cells in CC (Figure 7A). These morphological features indicate that the caspase-3 staining may locate at extracellular space or in the cytoplasm, which are consistent with those of cleaved caspase-3 immunofluorescent staining described in previous studies (Wang et al., 2015; Glushakov et al., 2018). Statistical analysis revealed that CPZ group had lower densities of labeled cells (including caspase-3, APC, and double-labeled cells) compared to CNT group (Figure 7B). The percentage of caspase-3 labeled cells in the APC positive cells in CPZ group, however, was significantly higher than that in CNT group (Figure 7C).

### Comparisons on Vulnerabilities of Brain Cells to CPZ-Induced Oxidative Stress

As shown in Table 2, CPZ exposure for 7 days dramatically decreased the density of APC positive cells in CC of mice compared to CNT group, but the treatment did not change the density of NeuN positive cells in PFC of mice. In contrast, the CPZ treatment paradigm increased densities of GFAP and iba-1 positive cells in CC of mice. These results suggest that OLs are most vulnerable to CPZ-induced oxidative stress, whereas neurons are most resilient to the insult though they are most susceptible to the intoxication. Increased numbers of astrocytes and microglia indicate the presence of compensatory reactions in these cells in response to CPZ intoxication.

**TABLE 2:** Differential vulnerabilities of brain cells to CPZ intoxication.

	APC^+^ cells	Iba-1^+^ cells	GFAP^+^ cells	NeuN^+^ cells
CNT	1076 ± 140	197 ± 38	469 ± 123	1730 ± 205
CPZ	327 ± 67	254 ± 57	658 ± 213	1744 ± 220
*P*	<0.0001	=0.063	<0.01	=0.791

*Data were expressed as mean ± SD (n/mm^2^). n = 6 per group. P-values resulted from Student t-test.*

## Discussion

CPZ has been shown to impair mitochondrial functions via acting on Complex IV of electron transport chain and antioxidant systems in mitochondria leading to ATP production deficit and oxidative stress (Ljutakova and Russanov, 1985; Acs et al., 2013; Kashani et al., 2014; Faizi et al., 2016; Scheld et al., 2019). The present study applied ^1^H-MRS method to measure mitochondrial function in brain cells of living mice and revealed lower levels of NAA and PCr in PFC and CPU of CPZ-exposed mice relative to controls. This result indicates that CPZ exposure for 7 days induced mitochondrial dysfunction in brain cells of the examined brain regions of mice as both NAA and PCr are produced in mitochondria of brain cells. Specifically, Of the two brain metabolites, NAA is synthesized in neuronal mitochondria (Baslow, 2000) and PCr serves as a rapidly mobilizable reserve of high-energy phosphates to recycle ATP in cells (Jacobus and Saks, 1982). This mitochondrial dysfunction was confirmed by biochemical analysis showing significantly lower ATP levels in the same brain regions of CPZ-exposed mice. Furthermore, we did *in situ* assessment of oxidative stress in brain cells by means of double immunofluorescent staining using cell-specific antibodies and that to 8-OHdG, which is regarded as a biomarker for oxidative damage to mtDNA. The data resulted from the application of this approach enable us to compare the susceptibility of each type of brain cells, including neurons, astrocytes, microglia, and OLs, to CPZ-induced mitochondrial dysfunction. As expected, OLs were shown to be more sensitive to the CPZ-induced mitochondrial dysfunction compared to astrocytes and microglia (Table 1). Interestingly, neurons in PFC and hippocampus of CPZ-exposed mice showed the highest percentage of 8-OHdG labeled cells in the NeuN positive cells, suggesting that neurons are most sensitive to CPZ-induced oxidative stress.

The concomitant presence of decreased NAA, PCr, and ATP in CPZ-exposed mice along with 8-OHdG positive cells strongly support that 8-OHdG is a marker of mtDNA damage in this study. Further evidence supporting this claim is the double immunofluorescent labeling of hippocampal neurons (CA3 area) by anti-NeuN and anti-8-OHdG (Figure 6). NeuN positive neurons showed intense staining of large nuclei, indicative of mature neurons. 8-OHdG positive staining profiles appeared cytoplasmic. In the merged photographs, the distinct localization of NeuN in nucleus and 8-OHdG in cytoplasm of neurons was further confirmed, indicating that 8-OHdG labeling occurred in mtDNA. Although oxidative damage may happen to nuclear DNA (nDNA), it can be repaired by means of 8-OHdG removal. However, mtDNA repairing mechanisms work less efficiently thus resulting in a 5–15 times higher mutation rate compared with nDNA (Lee and Wei, 2000; Payne et al., 2013).

That neurons are most sensitive to CPZ-induced oxidative stress is supported by an unexpected finding, i.e., mice in CNT group also showed NeuN/8-OHdG double labeled cells in hippocampus and PFC. This is in line with the previous finding of a significant increase in 8-OHdG immunoreactivity in the cytoplasm of hippocampal neurons but little to no reactivity in glia of patients with Alzheimer’s disease in which oxidative stress is an important player (Nunomura et al., 1999). To explain this seemly strange finding, we speculated that 8-OHdG positive cells in PFC and hippocampus of CNT group mice resulted from oxidative stress due to hypoxia, which had been induced by anesthesia and persisted into the subsequent surgical operation and perfusion phases. In line with this speculation, a recent animal study showed that the brains of all control animals (fetal sheep) expressed a degree of oxidative stress-induced DNA damage (8-OHdG positive cells) (Yawno et al., 2017). The anesthesia-related hypoxia and oxidative stress damage to mtDNA, however, does not mean that CPZ-exposure made no contribution to the oxidative stress seen in brain cells of CPZ-exposed mice. In support of this notion, CPZ-exposed mice presented lower levels of NAA and PCr in PFC and CPU relative to controls as described in the results of ^1^H-MRS experiment, in which mice were initially anesthetized with 5% isoflurane in oxygen and subsequently maintained with 1.5–2% isoflurane in oxygen delivered via a mask. This procedure may minimize the anesthesia-induced hypoxia.

Another finding in this study is that CPZ exposure for 7 days dramatically decreased the number of middle and large GST-pi positive cells, but significantly increased the number of small GST-pi positive cells in CC of mice. We believe that the small cells are immature OLs with GST-pi in the nucleus [GST-pi (Nuc)]; whereas middle and large ones are mature OLs with GST-pi in cytoplasm [GST-pi (Cyto)]. In line with this interpretation, GST-pi is initially located in the nucleus of NG2-positive OPCs. When OPCs lose NG2 immunoreactivity and differentiate into immature OLs, GST-pi gradually translocates to the cytoplasm (Tamura et al., 2007). Therefore, the CPZ-induced decrease in middle and large GST-pi positive cells is indicative of mature OLs loss whereas increased GST-pi positive small cells suggest an adaptive proliferation of immature OLs in response to CPZ-induced oxidative stress. To test this hypothesis, the immature OLs may be labeled with specific antibody like anti-OLIG2 in future experiments.

In line with the mature OLs decrease in CC of CPZ mice, CPZ exposure for 7 days significantly decreased the number of APC positive cells compared to CNT group. Relevantly, a previous study demonstrated that OLs death occurs very early (4 days after the initiation of CPZ diet) and precedes demyelination by weeks. And the study showed that the majority of dying OLs expressed high levels of activated caspase-3 on days 6 and 10, indicating an activation of the apoptosis cascade in OLs early during CPZ treatment (Hesse et al., 2010). Interestingly, the cell densities of caspase-3 positive cells and double labeled (caspase-3 and APC) cells in CPZ group in the present study were also significantly lower than those in CNT group. This may seem illogical at first, but it is not surprising considering the fact that mice in CNT group suffered hypoxia for a relatively long period which could impair mitochondrial function of mature OLs alike to what happened to neurons as discussed above. The anesthesia-induced hypoxia alone in CNT mice, however, was unable to cause such a severe damage to mitochondria as that in CPZ group leading to cell death. In other words, the caspase-3 positive cells in mice of CNT group may survive the anesthesia-induced hypoxia; in CPZ-treated mice, the same anesthesia-induced hypoxia aggravated the CPZ-induced oxidative stress thus may expedite the apoptosis process to the end stage. The dead cells were unlikely to be detected by immunofluorescent staining with the antibodies to caspase-3 and APC. All these facts and established knowledge explained why CPZ-exposed mice showed lower cell densities of caspase-3, APC, and double labeled cells compared to CNT group. The higher percentage of caspase-3 labeled cells in APC positive cells in CPZ group, however, resulted from a lower number of APC positive cells subsequent to continuous death of mature OLs.

Interestingly, immunofluorescent staining with the antibody to caspase-3 showed no apoptotic cells in PFC and hippocampus of both CNT and CPZ mice, suggesting the absence of apoptotic cells in the brain regions. How can be neurons most sensitive, but least vulnerable, to CPZ-induced oxidative stress? To answer this question, we reviewed relevant literature and summarized the pertinent information as follows: neurons are large cells relative to glial cells in the brain. Both the cell body and processes of neurons are filled with mitochondria at variable sizes; whereas the processes of glial cells have few or no mitochondria because these processes are too small/narrow to accommodate mitochondria. While fulfilling their high demand of energy supply necessary for specialized functions, including membrane ionic pumps, channel activity, and synaptic transmission, the high density of mitochondria in neurons enable them to be very sensitive to oxidative stress. Furthermore, mitochondria exhibit excess metabolic capacity that exceeds what is required for normal basic function as evidenced by the multi copy number of mtDNA serving as a structural excess capacity (Atamna et al., 2018). Moreover, complex IV exists in excess relative to the other three complexes in the electron transport chain (Davey and Clark, 1996). All these excess capacities in mitochondria may contribute to the highest resistance of neurons to CPZ-induced oxidative stress compared to glial cells containing fewer mitochondria as mentioned above.

Like that in small (immature) OLs, the cell densities of microglia and astrocytes significantly increased in CPZ-exposed mice compared to CNT group, suggesting that all these cells operate their proliferation capacity in response to CPZ-induced oxidative stress. Clearly, immature OLs proliferation prepared for remyelination that occurred simultaneously with or after demyelination in CPZ-exposed mice (Matsushima and Morell, 2001); whereas the microglia activation may exacerbate OLs death and demyelination via releasing pro-inflammation cytokines such as IL-1β, IL-6, and TNF-α (Aryanpour et al., 2017; Zhang et al., 2018). But, this does not necessarily mean that the mature OLs loss was initially caused by microglial activation or proinflammation cytokines released from activated microglia in this study. Instead, mature OLs loss may initiate microglia activation given that (1) OLs showed a highest vulnerability to CPZ-induced mtDNA damage as discussed above; (2) these cells have a reduced capacity to repair their mtDNA damage in comparison to other cell populations, possibly due to particularities in the expression of factors involved in DNA repairing mechanisms (Hollensworth et al., 2000); (3) accumulation of mutations within mtDNA is considered a factor leading to OLs stress, impairing mitochondrial functions, and increasing levels of ROS (Wang et al., 2013); and (4) conditioned medium of stressed OLs triggered microglia activation in a most recent study (Scheld et al., 2019).

In conclusion, CPZ exposure for 7 days induced mitochondrial dysfunction which in turn led to oxidative damage to mtDNA in brain cells of mice. To this CPZ-induced mitochondrial dysfunction, neurons in PFC are the most susceptible, followed by OLs, microglia, and astrocytes in the order, in terms of the proportion of 8-OHdG labeled cells in each type of these cells. In respect to the vulnerability of the brain cells to the CPZ-induced mitochondrial dysfunction, neurons are the most resilient cells as neuronal mitochondria have much excess metabolic capacities in energy and redox systems; whereas OLs are the most severely affected cells due to the low glutathione and high iron content, as well as the high oxidative metabolism because of the elevated production of membrane (Thorburne and Juurlink, 1996). Immature OLs, microglia, and astrocytes showed adaptive changes of proliferation and activation in response to CPZ-induced mitochondrial dysfunction. These changes are of help in protecting neurons against the CPZ-induced mitochondrial dysfunction in the early stage and may affect remyelination process in the late stage of the CPZ intoxication.

## Data Availability Statement

The datasets generated for this study are available on request to the corresponding author.

## Ethics Statement

The animal study was reviewed and approved by the Animal Use and Care Committee of Shantou University Medical College, in accordance with European Community’s Council Directive 2010/63/UE.

## Author Contributions

HX and SH designed the study and revised the manuscript. ML, MD, YZ, SX, and ZY conducted the study. ML and MD collected the data. ML, MD, and HX analyzed the data and drafted the manuscript. HX and MD interpreted the data. All the authors have read and approved the final version of the submitted manuscript.

## Conflict of Interest

The authors declare that the research was conducted in the absence of any commercial or financial relationships that could be construed as a potential conflict of interest.
